# Representing antibiotic relationships using measurements of efficacy against clinical isolates

**DOI:** 10.12688/wellcomeopenres.15304.3

**Published:** 2020-09-17

**Authors:** Liam Shaw

**Affiliations:** 1 Nuffield Department of Medicine, University of Oxford, Oxford, UK

**Keywords:** Antimicrobial resistance, AMR, data reuse, AMR surveillance, antibiotics, cross-resistance, chemical similarity

## Abstract

**Introduction.** Antimicrobial resistance (AMR) is a worrying and confusing problem for both patients and medical professionals. It is often difficult for non-specialists to understand how different antibiotics are related to one another. Here, I use experimental data from hundreds of thousands of clinical isolates to infer relationships between antibiotics and represent them with simple diagrams.

**Methods.** The minimum inhibitory concentration (MIC) of a bacterial isolate for a given antibiotic is defined as the lowest concentration that prevents visible growth. Measuring MICs for multiple antibiotics using the same isolate implicitly records the relationships of the antibiotics for a given species. The basic principle is that antibiotics with similar mechanisms of action should give rise to similar mechanisms of resistance, so should have correlated MICs across large numbers of isolates. This information can then be used to calculate distances between antibiotics based on pairwise correlations of their rank-ordered MICs. I apply this approach to a large historical AMR surveillance dataset (the Pfizer ATLAS surveillance dataset, 2004-2017).

**Results.** I demonstrate that clustering antibiotics in this way allows a simple visual comparison of how similar antibiotics are to each other based on their efficacy within a species. The resulting visualizations broadly recapitulate antibiotic classes. They also clearly show the dramatic effects of combining beta-lactam antibiotics with beta-lactamase inhibitors, as well as highlighting antibiotics which have unexpected correlations in MICs that are not predicted from their chemical similarities alone.

**Conclusion.** Large AMR surveillance datasets can be used in a hypothesis-free manner to show relationships between antibiotics based on their real-world efficacy.

## Introduction

Antimicrobial resistance (AMR) is a set of relationships. A bacterium is not simply ‘resistant’; it is resistant to an antibiotic. The problem of AMR is therefore an umbrella term for these many different pairwise relationships, each relationship specific to particular bacteria and particular antibiotics. We need approaches to represent the considerable complexity of these relationships in an understandable way.

It is undeniable that we need new antibiotics to treat patients with multi-drug-resistant infections ^1^; we have arguably also failed to keep track of or use existing antibiotics properly. A major driver of AMR is the inappropriate use of antibiotics. One meta-analysis estimated the pooled rate of inappropriate empirical treatment across available studies at 25.5–31.8% ^2^. Even recommended treatment is remarkably varied. For example, one survey found a diversity of recommendations for common conditions such as pneumonia across 105 NHS Trusts in England, in both the antimicrobial agent and the length of the treatment ^3^. Surprisingly little evidence is also available for the claim that failing to complete the full length of a physician-recommended course of antibiotics leads to the development of resistance ^4^.

These examples demonstrate that despite being conceptually simple (‘antibiotic use leads to resistance’), we currently lack a great deal of knowledge about AMR. Unfortunately, it seems that even having an awareness of existing knowledge about how different antibiotics relate to each other, and of the bacterial mechanisms which confer resistance, is challenging. A survey of Master of Pharmacy students at the University of Brighton, UK, found that around a third of third- and fourth-year students incorrectly agreed with a statement that ‘bacterial beta-lactamase enzymes inactivate aminoglycoside antibiotics’ ^5^. If future pharmacists can be so poorly taught about the mechanisms of AMR, what hope is there for the rest of us?

It is not an original observation that communicating AMR is challenging. Suggestions for improving this tend to focus around discourse, recommending caution about the use of catastrophist terms like ‘post-antibiotic apocalypse’ ^6^, moving away from the metaphor of a ‘war’ on ‘superbugs’ ^7^, or introducing the concept of an ‘antibiotic footprint’ analagous to a carbon footprint ^8^. The problem I seek to address here is not as general. I am specifically interested in the problem of imparting knowledge: how to convey the relationships between different antibiotics to non-specialists.

In order to do this, I use a large open dataset of experimental measurements on bacterial isolates taken from clinical settings. This dataset offers the opportunity to derive relationships between antibiotics that do not rely on knowledge of the underlying pharmacology, but are based on their actual efficacy. Simple visual representations of these show the similarities of antibiotics, giving an intuitive handle on the complexity of the relationships between them.

### Dataset

The Antimicrobial Testing Leadership and Surveillance (ATLAS) dataset was made publicly available as part of the Wellcome Data Re-use Prize ^9^. The ATLAS dataset comes from Pfizer’s overall programme to fulfill regulatory requirements and support appropriate use measures for both marketed antibiotics and antibiotics in development. It contains 633,820 isolates from 73 countries between 2004 and 2017. Each isolate has minimum inhibitory concentrations measured against multiple antibiotics (median 11, range: 5–23). For further details on the dataset and previous publications, see the ATLAS website ^10^.

Here, I report results for only the five most represented bacterial species in the dataset: *Staphylococcus aureus* ( *N* = 113,693); *Escherichia coli* ( *N* = 80,500); *Klebsiella pneumoniae* ( *N* = 64,296); *Pseudomonas aeruginosa* ( *N* = 61,799); and *Enterobacter cloacae* ( *N* = 39,391). Analysis includes all years and countries.

## Methods

This article developed from a submission to the Wellcome Data Re-use Prize (AMR surveillance). The original submission contains further code and analyses ^11^. Supplementary material for this article is available as *Extended data* on Figshare ^12^.

### Understanding minimum inhitbitory concentrations (MICs)

The ATLAS dataset contains information on the MIC of isolates tested against specific antibiotics. The MIC is the lowest concentration of an antibiotic that prevents visible growth. Consider the following table of isolate information:

**Table:** 

Isolate	Species	*A*_1_	*A*_2_	*A*_3_
*X*	*E. coli*	1	<0.5	4
*Y*	*E. coli*	2	<0.5	8

This information tells us that isolate *X* is an *E. coli* strain which cannot grow in concentrations of *A*_1_ of 1 mg/L, concentrations of *A*_2_ of 0.5 mg/L or more, and concentrations of *A*_3_ of 4 mg/L. Here, we are interested in the correlation between antibiotic MICs. For example, *A*_1_ and *A*_3_ appear to be correlated (on the basis of two isolates), because when the MIC for *A*_1_ halves so does the MIC of *A*_3_.

MICs are most commonly used to determine whether a bacteria is to be considered as ‘resistant’ to an antibiotic or not; an MIC is chosen based on clinical, experimental, and/or epidemiological data to mark the boundary between ‘susceptible’ and ‘resistant’ bacteria. Most publications on AMR report only resistance rates for single antibiotics. Sometimes, cross-resistance rates are considered (when a bacteria is resistant to two antibiotics at the same time). However, this approach still loses information from the full MIC data. Using the correlation of actual MIC values between pairs of antibiotics, and performing the analysis on thousands of isolates for all available antibiotics, should give us a definition of how similar antibiotics are based on their measured activities on real-world strains.

MICs are slightly unwieldy and complex variables to analyse statistically because they have unusual properties. For example, consider the MIC scale:

<1,1,2,4,8,>8

These values are ‘interval-censored’: the continuum of underlying true MICs is restricted by measurement into a scale of intervals, which contain ranges of values.

**Example:** if a concentration of 2 did not inhibit growth but a concentration of 4 did, the isolate has a ‘true’ MIC of *x* where 2 ≤ *x* < 4, but will have a recorded MIC of 4.

Also, the maximum value of the scale is ‘left-censored’: an MIC of >8 gives no information about the ‘true’ MIC apart from that it is greater than 8. Correspondingly, the minimum value is ‘right-censored’.

Approaches such as regression models that account for interval-censored data ^13^ have been applied to model MICs (e.g. 14). However, here I will use a naive and simple ‘solution’: I simply ignore the censoring. While not optimal, this is adequate for looking at large-scale correlations of MICs at a global level.

### MIC-based similarity scores

First, I order all possible values of MICs across the entire dataset. This step requires making an arbitrary choice about how to treat left/right-censored intervals i.e. those which feature an inequality. Here, I make a particular ordering choice which is best explained with an example of ordered MIC values:

<0.5,<1,1,>1,2,...,etc.

The reason that this is not ideal is that it assumes that '>1' is less than '2', but clearly a value of 2.1 could be ranked as '>1' in one scale and '2' in another. Thus, it is possible that identical isolates could be tested in different scales and ranked differently. Fortunately, the standardized methodology used in the ATLAS dataset means that for a given antibiotic, the scales are usually consistent, i.e. they will not contain potentially contradictory values. Even the subset which do, this should not qualitatively affect the rank correlations as it amounts to swapping (or removing) particular ranks.

**Figure 1.**
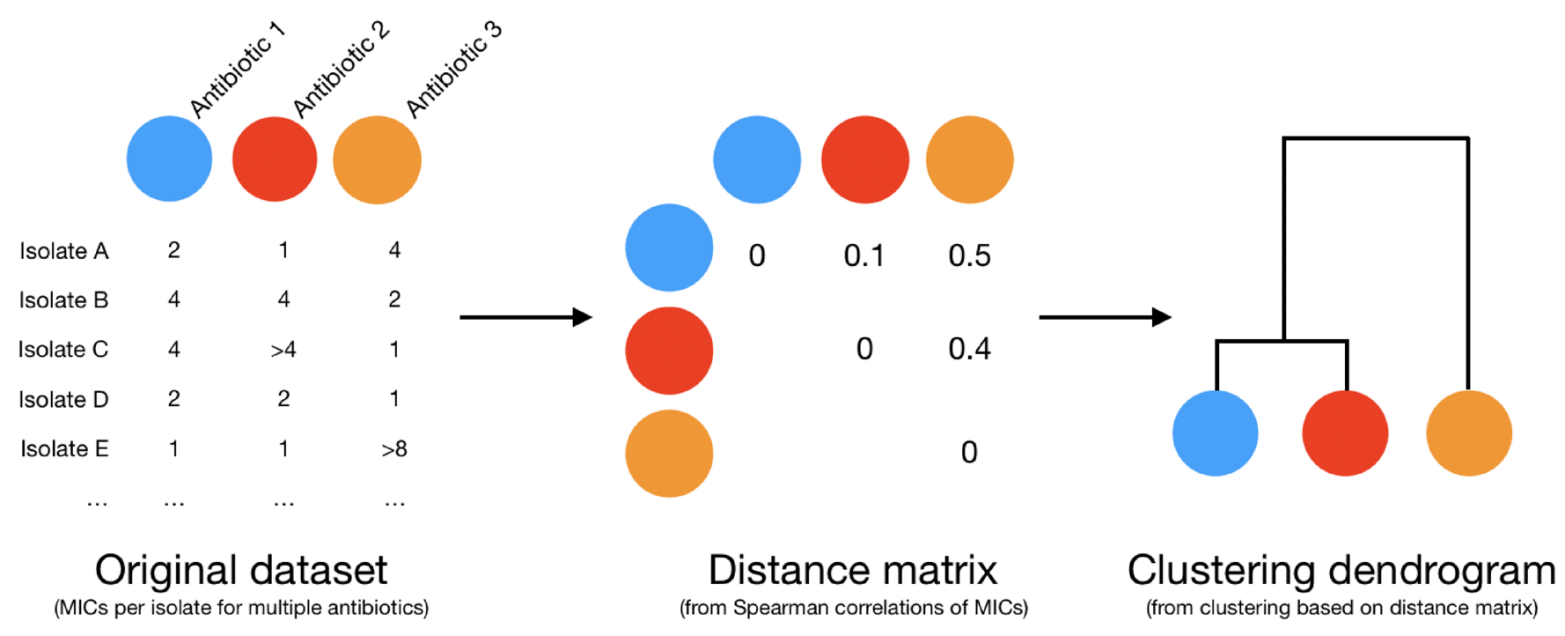
Schematic for antibiotic MIC correlation method. All possible pairwise correlations of MICs for pairs of antibiotics are calculated, and these are used to build a distance matrix and then a clustering dendrogram. Antibiotic 1 and Antibiotic 2 are strongly correlated (see dataset on the left), so have a smaller distance between them (0.1) than Antibiotic 1 and Antibiotic 3 (0.5).

Once MICs have been ranked, these ranked MIC values can then be used to calculate a Spearman rank correlation between antibiotics for a species. Figure 1 shows an overview of the procedure. Not all isolates are tested against all antibiotics, so the Spearman rank correlation *ρ*( *A*_1_, *A*_2_) between the MICs for antibiotics *A*_1_ and *A*_2_ is calculated only on the subset of isolates with measured MICs for both *A*_1_ and *A*_2_. Missing entries in the correlation matrix (where the antibiotics have never been tested simultaneously on any isolates of the species) are set to *ρ* = 0. These pairwise correlations are then used to create a distance matrix *d*( *A*_1_, *A*_2_) = 1 *− ρ*( *A*_1_, *A*_2_) ^2^ (see below). The distance matrix is then used to cluster antibiotics into a dendrogram with hclust in R (v3.5.1) ^15^ Ward’s hierarchical grouping algorithm ^16^. Dendrograms are plotted with ggdendro (v0.1-20) ^17^ and ggplot2 (v3.1.0) ^18^.

### Antibiotic classes

I obtained classification codes for each antibiotic by searching in the WHO ATC/DDD index 2019 ^19^. The Anatomical Therapeutic Chemical (ATC) Classification System gives a hierarchical classification of drugs according to their therapeutic properties. For example, ciprofloxacin has the code J01MA02, which should be read as:

$$\begin{array}{l} J\;\text{Anti-infectives}\;\text{for}\;\text{systemic}\;\text{use}\\ \to \;J01\;\mathrm{Antibacterials}\;\mathrm{for}\;\mathrm{systemic}\;\mathrm{use}\\ \;\to J01M\;\text{Quinolone}\;\text{antibacterials}\\ \;\to J01MA\;\mathrm{Fluoroquinolones}\\ \;\to J01MA02\;\mathrm{Ciprofloxacin} \end{array}$$

Some antibiotics in the ATLAS dataset are not yet classified by ATC. I classified combinations of J01D beta-lactams and avibactam as J01DI ('Other beta-lactams, Other').

### Chemical similarity scores

In order to compare the similarity scores generated from MICs in the ATLAS dataset, I also collected simplified molecular-input line-entry system (SMILES) representations for all single-molecule antibiotics (excluding combination antibiotics e.g. piperacillin/tazobactam) from their respective pages on Wikipedia (2nd May 2019). I then used the R package rcdk (v3.4.7.1) ^20^ and followed the instructional vignette ^21^ to parse these with parse.smiles, obtain the chemical fingerprint with get.fingerprint and then construct a distance matrix with fp.sim.matrix using the Tanimoto similarity metric (in a quirk of disciplines, this is the same as the – to me – more familiar Jaccard coefficient ^22^). This gives a score for each pair of antibiotics based on how many chemical features they share.

For ten single-molecule antibiotics that had >5,000 measurements for each of the top five bacterial species (ampicillin, ceftaroline, ceftazidime, ceftriaxone, doripenem, imipenem, levofloxacin, meropenem, minocycline, tigecycline) I calculated the correlation between the chemical similarity matrix and the MIC-based similarity matrix using mantel.randtest from the R package ade4 (v1.7-13) ^23^. Table 1 shows that the strength of the correlation was increased for all species when the MIC-based Spearman correlation was squared, so I used this to produce the MIC-based distance matrix between antibiotics.

**Table 1.** Mantel correlations between chemical similarities and MIC-based similarities using linear ( *ρ*) or squared ( *ρ* ^2^) Spearman correlations, for the ten antibiotics with >5,000 measurements for each of the top five bacterial species. There is a significant strong association between the similarities. Squaring the MIC-based correlations increases the strength of the correlation with chemical similarities. *M* is the observed correlation between distance matrices using the Mantel test, *p* is the p-value.

	*ρ*		*ρ* ^2^	
Species	*M*	*p*	*M*	*p*
*S. aureus*	0.319	0.019	0.396	0.006
*E. coli*	0.516	0.001	0.541	0.001
*K. pneumoniae*	0.517	0.002	0.566	0.001
*P. aeruginosa*	0.602	0.001	0.729	0.001
*E. cloacae*	0.585	0.001	0.672	0.001

## Results

**Figure 2.**
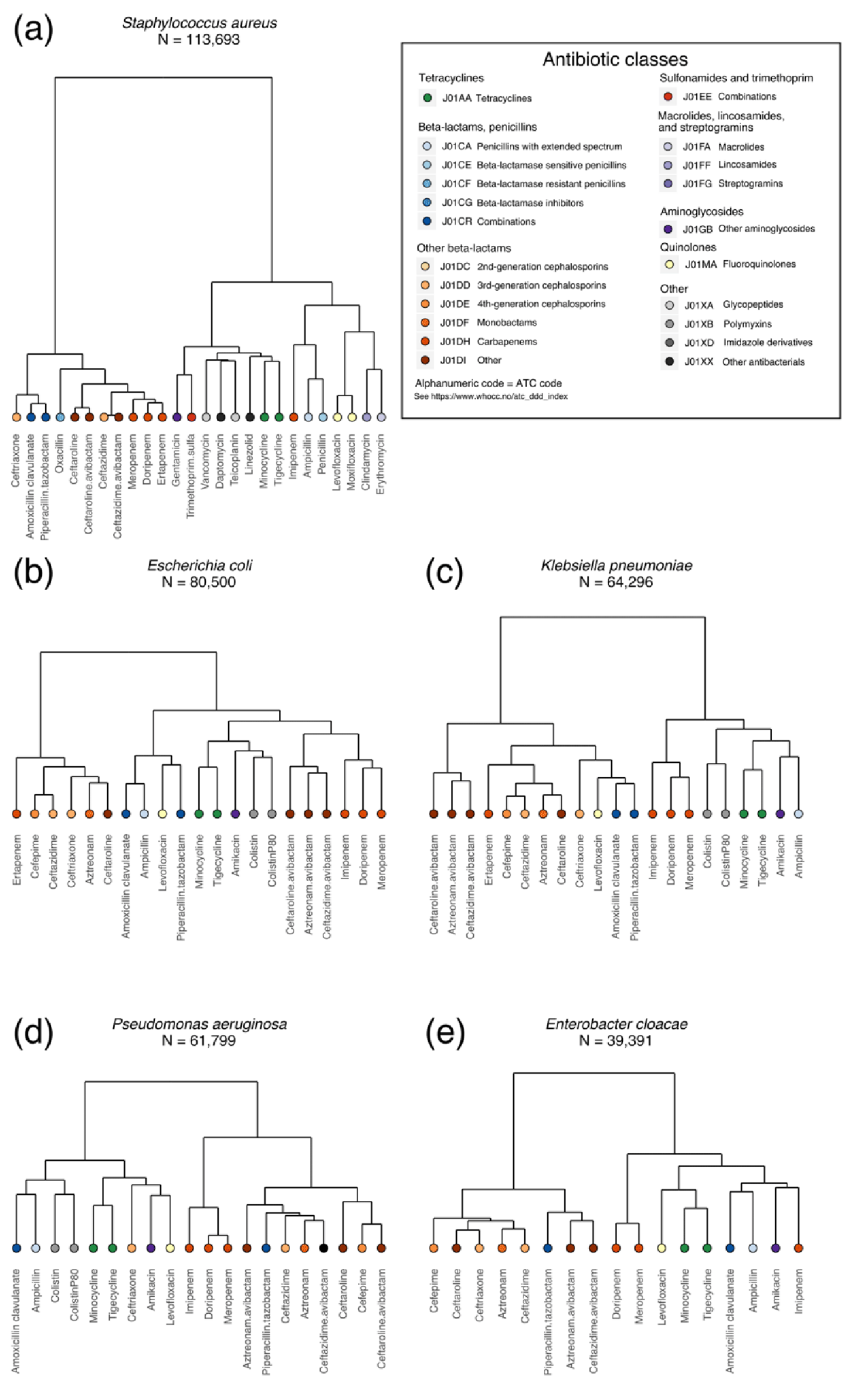
Clustering of antibiotics according to their MIC correlations within the top five species in the ATLAS dataset. Similar antibiotics generally cluster together across all five species ( **a**)–( **e**). However, the precise pattern of this clustering varies by species. Colour of points indicates the class of antibiotic, as judged using the WHO ATC code for the drug (see legend, top right). Greater vertical distance represents greater separation (vertical axis conserved across subfigures). Only antibiotics with >5,000 test results for a species are included. Total number of isolates is shown in title of each subfigure.

Clustering antibiotics based on pairwise distances calculated from correlations of their MICs broadly captures known antibiotic classes ( Figure 2). The layout of a dendrogram gives a clear visualisation of when antibiotics are very similar in terms of their efficacy on clinical isolates of a bacterial species. They also allow a quick comparison of antibiotics in terms of their action across different species.

Although the whole point of these visualisations is to convey a general overview of the relationships from the dataset alone, independent of any previous pharmacological knowledge, it is perhaps worth pointing out a few examples of how they connect to (and convey) what is known about AMR.

**Similarities within antibiotic classes.** Tigecycline and minocycline (both members of the tetracycline class) cluster together in all five species. This is unsurprising, as tetracylines all have the same mechanism of action: they bind to 16S ribosomal RNA (rRNA) in the ribosome and thus inhibit protein synthesis ^24^. Mutations in the 16S rRNA gene which confer resistance to e.g. tigecycline typically also do so for all tetracyclines.

**Similarities across antibiotic classes.** Ampicillin and amoxicillin clavulanate are clearly very similar in *E. coli* ( Figure 2b). However, the community usage of the J01CA subclass of antibiotics (which includes ampicillin) across 12 European countries does not correlate well with the prevalence of resistance to ampicillin ^11^. This might seem surprising, but even without the pharmacological knowledge that ampicillin and amoxicillin are very similar in activity we could use this clustering of antibiotics to verify that amoxicillin clavulanate usage (in the J01CR class) correlates with ampicillin resistance ^11^. This underlines the importance of being aware of the mechanisms of AMR rather than just fitting statistical models to data.

**Species-specific effects.** The beta-lactam antibiotic ceftazidime has limited activity against *S. aureus*. Therefore, combining it with avibactam (a non-beta-lactam beta-lactamase inhibitor with little to no antibiotic activity of its own) has little additional effect; the two treatments cluster close together ( Figure 2a). However, the dramatic effect of using avibactam in combination with ceftazidime in Gram-negative species such as *E. coli* and *K. pneumoniae* is readily apparent; ceftazidime/avibactam is shown far apart from ceftazidime in these species ( Figure 2b–2e).

## Discussion

I have outlined how a large AMR surveillance dataset can be used to characterise relationships between antibiotics with no prior knowledge of pharmacology. I have shown that computing distances between antibiotics based on their correlations across clinical isolates shows a good correlation with chemical similarity scores ( Table 1) and can be used to produce clear visualisations of relationships in AMR ( Figure 2).

These visualisation make clear one of the challenges of AMR: use of one antibiotic can lead to increasing resistance to other antibiotics as well due to their chemical similarity. By representing antibiotics closer together when their MICs are correlated, the diagrams convey the fact that bacteria increasing their MIC to one specific antibiotic can be associated with an increased MIC for other similar antibiotics. Whole branches of the tree may become ‘off-limits’ once resistance develops, rather than antibiotics being picked off one by one. Speculatively, simple displays based on similar visualisations could be used in multiple settings: when explaining the importance of antibiotic stewardship in hospitals; when recommending courses of antibiotics for patients; or even included on antibiotic packaging to underline the difference between antibiotics and other classes of drugs. However, I want to make clear I don't think the figures in this paper are currently fit for this purpose.

### Limitations

As noted, I have not attempted to perform any serious statistical treatment of the MICs and have simply sorted them into rank-order, acknowledging that this can lead to inconsistent measured MICs from identical true MICs. It is possibly that improving this step could improve the quality of the inferred relationships. Furthermore, aside from a crude attempt to check broad consistency with chemical similarity scores, I have not attempted to prove that these results have strong meaning beyond simply representing the data. Because of the fact that not all antibiotics are tested against all others, specific pairwise comparisons may have little meaning, and the broad structure of the dendrogram may be driven by a biased pattern of testing.

This method implicitly assumes a ‘one size fits all’ approach for a given species. In reality, there are typically multiple mechanisms of resistance to antibiotics. For the beta-lactams, over 2,000 separate resistance genes are known. Furthermore, high correlations between antibiotic MICs for a particular bacterial species could be driven by multiple factors. Possible scenarios include but are not limited to: the antibiotics have similar activity and therefore similar resistance mechanisms; the antibiotics are commonly used together, so because resistances are selected at the same time they are commonly associated despite different resistance mechanisms; certain strains possess multiple mechanisms of drug-resistance and drive the high correlation. I have not attempted to disentangle these causes here.

I caution against inferring potential treatment interactions between antibiotics based on these dendrograms. In general, two antibiotics used in combination can be additive, synergistic (i.e. greater than the sum of their individual effects), or antagonistic (i.e. less than the sum of their individual effects). Phenotypic testing of combinations is necessary to understand these interactions.

Even leaving this point aside, the clinical relevance of what I have done is unclear. The term ‘resistance’ is tied to a specific clinical meaning: that standard treatment at recommended doses will likely not be effective in a patient infected with such a strain. The MIC offers a proxy for determining when this is true, but is not in itself a clinical parameter. However, I would argue that using the whole range of the MIC distribution to produce the visualisations is more statistically justifiable to get an overview of the range of relationships between antibiotics. Correlations could be calculated based on binary cross-resistance proportions and equivalent diagrams plotted, but proportions of cross-resistant isolates may be very small.

Finally, the underlying data has its own limitations. Despite being of high quality and standardisation, MIC phenotypes can vary between experiments. For example, colistin adheres to plastics, so sometimes MICs are tested after adding the surfactant polysorbate 80 (P80), which can alter the MIC of Gram-negative species ^25^, as seen in the data here ( Figure 2b–d).

### Future work

This work is intended only as a sketch of a possible way to represent relationships between antibiotics using large datasets from clinical settings. I am sure the elementary approach I have outlined here could be improved upon; I offer some suggestions here.

As other large AMR historical surveillance datasets become available, as endorsed in principle by major pharmaceutical companies ^26^, correlations of quantitative MICs can also be investigated in these datasets. Furthermore, as sequencing becomes cheaper, it will become increasingly common to also perform whole genome sequencing of banks of isolates or do other molecular characterization. Linking MIC-based relationships to the genetic mechanisms of AMR would be an extension of this work.

During the course of preparing this article for publication, I have come across other authors noting the complementary information provided by ‘semantic similarity’ and ‘chemical similarity’. Notably, one such article’s illustrative example of two drugs that are chemically very different but semantically similar, is two beta-lactamase inhibitors ^27^. It is also true that compounds with a high chemical similarity score do not necessarily have the same biological activity ^22^. Combining the complementary information of chemical similarity scores with semantic ‘efficacy’ scores based on MICs could lead to a better representational hierarchy of existing antibiotics. Unfortunately, clinical efficacy scores by definition do not exist for novel compounds, so this would not help with drug development (a common use of chemical similarity scores).

At present, cross-resistance rates between major classes of antibiotics are reported in surveillance. It would in theory be possible to model antibiotic cross-correlation rather than simply cross-resistance. Comparing the cross-correlation distributions between different patient groups, geographical areas, or over time, would reveal additional insights. The problem I see here is defining what is ‘of interest’ as a substantially different correlation between two groups. Confidence intervals can be calculated on Spearman rank correlations (using the Fisher z-transformation), but because of the large numbers of isolates, even in subgroups, these would rarely overlap. Finding a ‘statistically significant’ difference between subgroups does not necessarily mean clinical or biological significance.

I have argued for the importance of a general overview of relationships in AMR, but the specifics are vital. There is still a great deal we still do not understand about the mechanisms of action and resistance for antibiotics. Novel cross-resistance phenotypes continue to challenge previously held ideas about the relationship of use of one antibiotic with resistance to another. For example, in a recent report of an unexpected mechanism conferring resistance to ceftazidime in *Stenotrophomonas maltophilia*, the authors conclude that using ceftazidime may already be ‘eroding the future potential’ of experimental siderophore-conjugated antibiotics against this species ^28^. With mechanisms such as this in mind, one option would be to use MIC-based correlations to quantify the strength of possible interactions between antibiotics in terms of shared resistance mechanisms. These values could then be used in subsequent modelling attempts to link antibiotic consumption rates to resistance rates.

## Conclusion

AMR surveillance data is a valuable source of information – not only for historical resistance rates for single antibiotics, but also because it contains information about the relationships between antibiotics. Visualising similarities between antibiotics based on their efficacy against real clinical isolates is one way to use this information.

## Data availability

### Underlying data

Access to the data was through the Wellcome Data Re-use Prize – Surveillance ^9^, Synapse ID syn17009517.

Data underlying the results of this study can be obtained from the ATLAS Programme, which was run by Pfizer. Data are available from https://atlas-surveillance.com, following free registration.

### Extended data

Figshare: Representing antibiotic relationships using measurements of efficacy against clinical isolates. https://doi.org/10.6084/m9.figshare.8159525.v1 ^12^.

The following extended data are available:

- **Supplementary File 1**. The ATLAS dataset after data cleaning and ordering of MICs.
- **Supplementary File 2**. Metadata on antibiotics (classes, ATC code, SMILES).
- **Supplementary File 3**. R code necessary to reproduce Table 1 and Figure 2.

Extended data are available under the terms of the Creative Commons Attribution 4.0 International license (CC-BY 4.0).
