# Supplementary material for: Representing antibiotic relationships using measurements of efficacy against clinical isolates
Source: Wellcome Open Res. 2020 Sep 17;4:86. Originally published 2019 May 28. [Version 3] doi: 10.12688/wellcomeopenres.15304.3 (PMC7520714; doi:10.12688/wellcomeopenres.15304.3)
Supplement: Peer review history

# Peer review: Reviewer response for version 3

**DOI:** 10.21956/wellcomeopenres.17924.r40499

**Published:** 2020-09-24

**Authors:** Benno H Ter Kuile

**Affiliations:** 1 Laboratory for Molecular Biology and Microbial Food Safety, University of Amsterdam, Amsterdam, The Netherlands

I am satisfied with the changes made by the author. I am not sure I would have written the same paper, but it remains his responsibility as an author and there are no obviously incorrect or unsupported statements in the present text.

Is the work clearly and accurately presented and does it cite the current literature?

Yes

If applicable, is the statistical analysis and its interpretation appropriate?

Not applicable

Are all the source data underlying the results available to ensure full reproducibility?

Yes

Is the study design appropriate and is the work technically sound?

Partly

Are the conclusions drawn adequately supported by the results?

Partly

Are sufficient details of methods and analysis provided to allow replication by others?

Yes

Reviewer Expertise:

Antimicrobial resistance, specifically mechanism of de novo development.

I confirm that I have read this submission and believe that I have an appropriate level of expertise to confirm that it is of an acceptable scientific standard.

# Peer review: Reviewer response for version 2

**DOI:** 10.21956/wellcomeopenres.16965.r39923

**Published:** 2020-08-27

**Authors:** Benno H Ter Kuile

**Affiliations:** 1 Laboratory for Molecular Biology and Microbial Food Safety, University of Amsterdam, Amsterdam, The Netherlands

This manuscript presents an innovative approach to the analysis of cross resistances for 5 bacterial species and 22 antibiotics. As such it makes a valuable contribution. I have, however, some reservations of a general nature that I hope can be be addressed.

 The author oversells the insights considerably. Statements such as: “Current antimicrobial nomenclature makes it difficult to understand how different antibiotics are related to one another, and their empirical uses and efficacy”, suggest more than is delivered. The results are five relationship trees comparable to evolutionary trees that show the probability that resistance for one antibiotic in one of the five species correlates to resistance for another. Again, the approach is valuable, but it does not solve the problems the author mentions, such as misconceptions of third and fourth years pharmacy students. If the clinical relevance is unclear (see page 7), then one should avoid sweeping statements.

 The interpretation of Table 1 does not square very well with Figure 2. While it is claimed that chemical similarity and resistance correlate, this is not obvious from Figure 2. For the analysis of Table 1, ten antibiotics were selected. Was this selection random, or were these the ten that gave the anticipated outcome?

 All in all, I am not convinced that the last sentence of the conclusion is justified by the preceding text. I suggest the author takes a close look at it before taking responsibility for it.

Is the work clearly and accurately presented and does it cite the current literature?

Yes

If applicable, is the statistical analysis and its interpretation appropriate?

Not applicable

Are all the source data underlying the results available to ensure full reproducibility?

Yes

Is the study design appropriate and is the work technically sound?

Partly

Are the conclusions drawn adequately supported by the results?

Partly

Are sufficient details of methods and analysis provided to allow replication by others?

Yes

Reviewer Expertise:

Antimicrobial resistance, specifically mechanism of de novo development.

I confirm that I have read this submission and believe that I have an appropriate level of expertise to confirm that it is of an acceptable scientific standard, however I have significant reservations, as outlined above.

# Response

**Published:** 2020-09-09

**Authors:** Liam Shaw

**Affiliations:** University of Oxford, UK

*This manuscript presents an innovative approach to the analysis of cross resistances for 5 bacterial species and 22 antibiotics. As such it makes a valuable contribution. I have, however, some reservations of a general nature that I hope can be be addressed.*

Thanks for the fair and critical assessment.

*The author oversells the insights considerably. Statements such as: “Current antimicrobial nomenclature makes it difficult to understand how different antibiotics are related to one another, and their empirical uses and efficacy”, suggest more than is delivered. The results are five relationship trees comparable to evolutionary trees that show the probability that resistance for one antibiotic in one of the five species correlates to resistance for another. Again, the approach is valuable, but it does not solve the problems the author mentions, such as misconceptions of third and fourth years pharmacy students. If the clinical relevance is unclear (see page 7), then one should avoid sweeping statements.*

I have made a few alterations to try to avoid "overselling". For example, in the discussion I have made clear that the possible future uses of similar diagrams I suggest are very much speculation, and that I don't think the figures in this paper are currently fit for these purposes. I hope these changes are satisfactory.

*The interpretation of Table 1 does not square very well with Figure 2. While it is claimed that chemical similarity and resistance correlate, this is not obvious from Figure 2. For the analysis of Table 1, ten antibiotics were selected. Was this selection random, or were these the ten that gave the anticipated outcome?*

Apologies if this was unclear. These were the ten single-molecule antibiotics with the most data: they all had >5,000 measurements for each of the top five bacterial species. This information was in the text but was missing from the caption for Table 1. It has now been added. 

*All in all, I am not convinced that the last sentence of the conclusion is justified by the preceding text. I suggest the author takes a close look at it before taking responsibility for it.*

I acknowledge this criticism. I have deleted the last sentence of the conclusion in the abstract, and altered the last sentence of the conclusion: "Visualising similarities between antibiotics based on their efficacy against real clinical isolates is one way to use this information. "

# Peer review: Reviewer response for version 2

**DOI:** 10.21956/wellcomeopenres.16965.r36666

**Published:** 2019-10-18

**Authors:** Mark H. Wilcox

**Affiliations:** 1 University of Leeds, Leeds, UK

I am happy with the revisions.

Is the work clearly and accurately presented and does it cite the current literature?

Partly

If applicable, is the statistical analysis and its interpretation appropriate?

Partly

Are all the source data underlying the results available to ensure full reproducibility?

No source data required

Is the study design appropriate and is the work technically sound?

Partly

Are the conclusions drawn adequately supported by the results?

No

Are sufficient details of methods and analysis provided to allow replication by others?

Partly

Reviewer Expertise:

NA

I confirm that I have read this submission and believe that I have an appropriate level of expertise to confirm that it is of an acceptable scientific standard.

# Peer review: Reviewer response for version 1

**DOI:** 10.21956/wellcomeopenres.16704.r36348

**Published:** 2019-09-23

**Authors:** Mark H. Wilcox

**Affiliations:** 1 University of Leeds, Leeds, UK

This is an interesting manuscript. The premise (that ‘antibiotics with similar activities have similar mechanisms of action’) is only partly correct. Actually, two antibiotics with identical MICs for a given strain could act by completely different mechanisms.

 That the analysis shows ‘The resulting visualizations broadly recapitulate antibiotic classes’ is not surprising. The next claim that ‘They also clearly show the dramatic effects of combination therapies …’ is an important potential leap of faith. The author describes a theoretical effect of combining antibiotics that may not actually be true in reality, as the effects of two antibiotics may be additive, synergistic or antagonistic. The only way to determine which of these is true for any given combination of antibiotics and for a specific strain (or possibly species) is to carry out phenotypic testing (typically using a chequerboard techniques). The combinations of a β-lactam and a β-lactamase inhibitor (e.g. piperacillin-tazobactam) is a very specific scenario, which technically is not usually referred to a combining antibiotics; rather, it is using an antibiotic (e.g. piperacillin) plus an enzyme inhibitor (e.g. tazobactam). The enzyme inhibitor usually has no (or very little) antibiotic activity itself.

 As such, the manuscript/abstract is currently potentially misleading and requires modification.

Is the work clearly and accurately presented and does it cite the current literature?

Partly

If applicable, is the statistical analysis and its interpretation appropriate?

Partly

Are all the source data underlying the results available to ensure full reproducibility?

No source data required

Is the study design appropriate and is the work technically sound?

Partly

Are the conclusions drawn adequately supported by the results?

No

Are sufficient details of methods and analysis provided to allow replication by others?

Partly

Reviewer Expertise:

NA

I confirm that I have read this submission and believe that I have an appropriate level of expertise to confirm that it is of an acceptable scientific standard, however I have significant reservations, as outlined above.

# Response

**Published:** 2019-10-02

**Authors:** Liam Shaw

**Affiliations:** University of Oxford, UK

*This is an interesting manuscript. The premise (that ‘antibiotics with similar activities have similar mechanisms of action’) is only partly correct. Actually, two antibiotics with identical MICs for a given strain could act by completely different mechanisms.*

I agree with the last statement. The fuzzy premise of ‘similar activities’ against a given strain implying ‘similar mechanisms of action’ is indeed not necessarily true, and in fact is not necessary for the correlation approach – the idea is to look at the patterns across isolates, where covariation would be suggestive of similar (or linked) mechanisms of resistance. I have rewritten this statement in the abstract, which I hope better summarises the central idea:

"The basic principle is that antibiotics with similar mechanisms of action should give rise to similar (or linked) mechanisms of resistance, so should have correlated MICs across large numbers of isolates."

*That the analysis shows ‘The resulting visualizations broadly recapitulate antibiotic classes’ is not surprising. The next claim that ‘They also clearly show the dramatic effects of combination therapies …’ is an important potential leap of faith. The author describes a theoretical effect of combining antibiotics that may not actually be true in reality, as the effects of two antibiotics may be additive, synergistic or antagonistic. The only way to determine which of these is true for any given combination of antibiotics and for a specific strain (or possibly species) is to carry out phenotypic testing (typically using a chequerboard techniques).*

I agree the 'leap of faith' interpretation would be stretching it! I wasn’t intending to imply that the correlation dendrograms could be used to derive effects of combining antibiotics – which of course should be tested phenotypically. It arose from a simple misunderstanding on my part about what the term ‘combination therapy’ includes (see next comment). I have also added a paragraph to the discussion cautioning against any inference of potential effects of combining antibiotics from these dendrograms:

"I caution against inferring potential treatment interactions between antibiotics based on these dendrograms. In general, two antibiotics used in combination can be additive, synergistic (i.e. greater than the sum of their individual effects), or antagonistic (i.e. less than the sum of their individual effects). Phenotypic testing of combinations is necessary to understand these interactions."

*The combinations of a β-lactam and a β-lactamase inhibitor (e.g. piperacillin-tazobactam) is a very specific scenario, which technically is not usually referred to a combining antibiotics; rather, it is using an antibiotic (e.g. piperacillin) plus an enzyme inhibitor (e.g. tazobactam). The enzyme inhibitor usually has no (or very little) antibiotic activity itself.*

I was aware that in combinations such as piperacillin-tazobactam the enzyme inhibitor (tazobactam) has very little to no antibiotic activity itself. However, I wasn’t aware that referring to this as a ‘combination therapy’ would be misleading and invite extension of the statement to dual-antibiotic combinations. I have rewritten the abstract accordingly:

"They also clearly show the dramatic effects of combining beta-lactam antibiotics with beta-lactamase inhibitors, as well as highlighting antibiotics which have unexpected correlations in MICs that are not predicted from their chemical similarities alone."

I have also rewritten the appropriate section of the results to make clear that the combination beta-lactam and beta-lactamase inhibitor is a very specific scenario:

"The beta-lactam antibiotic ceftazidime has limited activity against *S. aureus*. Therefore, combining it with avibactam (a non-beta-lactam beta-lactamase inhibitor with little to no antibiotic activity of its own) has little additional effect; the two treatments cluster close together (Figure 2a). However, the dramatic effect of using avibactam in combination with ceftazidime in Gram-negative species such as *E. coli *and *K. pneumoniae *is readily apparent; ceftazidime/avibactam is shown far apart from ceftazidime in these species (Figure 2b–2e)."

*As such, the manuscript/abstract is currently potentially misleading and requires modification*

Thank you for highlighting these points. I hope the modifications are satisfactory and clear up any potential confusion or misunderstanding.
